# A decadal analysis of road traffic accident‐related mortality among women of reproductive age: A cross‐sectional study in East Azerbaijan Province, Iran

**DOI:** 10.1002/hsr2.2058

**Published:** 2024-05-06

**Authors:** Mina Golestani, Homayoun Sadeghi‐Bazargani, Leili Faraji Gavgani, Roghayeh Khabiri, Leila Jahangiry

**Affiliations:** ^1^ Road Traffic Injury Research Center Tabriz University of Medical Sciences Tabriz Iran; ^2^ Statistics and Epidemiology Department, Road Traffic Injury Research Center Tabriz University of Medical Sciences Tabriz Iran; ^3^ Statistics and Epidemiology Department Tabriz University of Medical Sciences Tabriz Iran; ^4^ Tabriz Health Services Management Research Center Tabriz University of Medical Sciences Tabriz Iran; ^5^ Department of Health Education and Health Promotion Tabriz University of Medical Sciences Tabriz Iran

**Keywords:** East Azerbaijan, epidemiology, reproductive age, road traffic injuries, women

## Abstract

**Background:**

Given the extensive impact of road traffic accidents (RTAs) consequences and their potential ramifications on the health of both current and future generations, this study examines the social and demographic factors that influence RTA‐related mortality among women of reproductive age.

**Methods:**

The study population consisted of cases retrieved from the database of the Legal Medicine Organization, encompassing all women aged 15–49 who succumbed to road accidents between 2011 and 2021.

**Results:**

The mean age of women in the reproductive age group from East Azerbaijan province between 2011 and 2021 was 33.67 years, with a standard deviation of 9.18. RTAs on main roads accounted for the majority of incidents (395 cases, 50.8%), with 93.7% (728 cases) attributed to road traffic. In 54.4% of these cases, the affected organ was the head and neck, and the primary cause of death in 52.1% was head trauma. Across all age groups, injuries to the neck and head were the most common, followed by injuries to the abdomen, chest, back, and sternum.

**Conclusion:**

The higher incidence of road accidents in the 25–29 age group highlights the need for targeted interventions to address risky behaviors, inexperience, and peer influences in this demographic. Our observation of passengers experiencing the highest mortality rate emphasizes the vulnerability of road users, particularly pedestrians, in traffic accidents. Pedestrian violations in the 17–30 age group further emphasize the importance of education and awareness campaigns aimed at reducing risky behavior.

## 1 INTRODUCTION

Road traffic accidents (RTAs) are the main health challenge and a main cause of mortality in Iran. The extent of RTAs‐related damages is notably broad in Iran and worldwide making it one of the main public health challenges.^1^ In all over the world including Europe, RTA‐related injuries are the main cause of mortality in the 15–29 age group and one of the five main cause of death in women of reproductive age (15–49 years old).^2^ Reproductive age has unique effects on women's health and welfare because they face a broad range of health challenges at this age that significantly affect the current and next generations.^3^ The multidimensional nature of RTAs has been confirmed in which different factors such as population density of the area, urbanization, vehicle and road infrastructure, safety issues, behavior, and environmental effects (related to prehospital and posthospital medical procedures) play a significant role in the mortality rate of RTAs.^4^ Besides overwhelming human mortality in adults of reproductive age, RTAs impose a heavy economic burden on human life.^5^ Predictions suggest that if immediate measures won't be taken, RTAs will ascend from eighth main cause of mortality to seventh with the low‐ and middle‐income countries (LMICs) as the key players in this ascend.^5^

Considering the importance of evaluating the main factors that increase human mortality, the current study aimed to evaluate social and demographic factors that affect RTA‐related mortality in women in reproductive age (aged 15–49) using data from the Legal Medicine Organization.

## 2 MATERIALS AND METHODS

### 2.1 Study population and data source

The current study utilized data from Legal Medicine Organization database about women of reproductive age (15–49 years) in the recent decade gathered by adopting total sampling method. Women in the reproductive ages (15–49 years) were defined based on the World Health Organization.^6^ The Legal Medicine Organization of East Azerbaijan Province covers data on mortality in East Azerbaijan Province. Iranian laws enforce that RTA‐related mortality cases happening even after 30 days postaccident should be assessed in Legal Medicine centers by autopsy. The centers record this mortality and the data are sent to the main center located in the city of Tabriz.^6^ After data retrieval, the invalid cases were excluded and the analysis was conducted.

### 2.2 Variables

The main outcome of the current study was to determine the distribution pattern of RTA‐related mortality among women in reproductive age (15–49 years) based on demographic features from 2011 to 2021 extracted from the Legal Medicine Organization database. The mortality rate of women in reproductive age was achieved based on the demographic features including age (divided into three age groups: 15–29, 30–39, and 40–49) and marital status (married, single, not answered), and independent variables including the type of accident (traffic, nontraffic), road user type (driver/rider, pedestrian, passenger), and affected organ (head and neck, chest and abdomen, back and sternum, shoulder joint down, and femur to the bottom).

### 2.3 Statistical analysis

Data were briefly presented in the form of descriptive statistics of number (percent). Invalid data were excluded and the final data were analyzed. The data were compared utilizing *χ* ^2^, Pearson, and Fisher's two‐sided statistical test (if needed). Confidence interval and *p* value were 95% and 0.05, respectively. STATA version 17 (STATA Corp.) was utilized for the data analysis.

## 3 RESULTS

### 3.1 Key demographic attributes of RTA fatalities among women of reproductive age

The mean age of women in the reproductive age group from East Azerbaijan province between 2011 and 2021 was 33.67 years, with a standard deviation of 9.18. Among these women, 284 cases (36.6%) fell within the 15–29 age group, 249 cases (32.0%) were in the 30–39 age group, and 244 cases (31.4%) were in the 40–49 age group. The majority of accidents (379 cases, 48.8%) involved vehicle collisions with other vehicles. RTAs on main roads accounted for the majority of incidents (395 cases, 50.8%), with 93.7% (728 cases) attributed to road traffic. In 54.4% of these cases, the affected organ was the head and neck, and the primary cause of death in 52.1% was head trauma. The majority of accidents (31.8%) occurred during the summer season, and only 6.9% of cases resulted in fatalities during transportation to the hospital. Table 1 provides an overview of mortality patterns related to RTAs in women of reproductive age, categorized by age groups and demographic variables, along with details about the accidents.

**Table 1:** The distribution of RTA‐related mortality in women of reproductive age based on age groups, demographics, and accident details in 2011–2021.

	Mortality, *n* (%)
Age (year)	
15–19	48 (6.18)
20–24	99 (12.74)
25–29	137 (17.63)
30–34	125 (16.0)
35–39	126 (16.2)
40–44	118 (15.19)
45–49	126 (16.22)
Education	
≤Diploma	615 (81.78)
>Diploma	137 (18.22)
Marital status	
Single	149 (19.20)
Married	619 (79.77)
Unknown	8 (1.03)
Employment status	
Unemployed	78 (10.0)
Employed	118 (15.23)
Housekeepers	569 (73.42)
Unknown	12 (1.29)
The manner of the accident	
Rollover	212 (27.3)
Vehicle fire	1 (0.1)
Vehicle	552 (71.0)
Other	12 (1.2)
Rode type	
Freeway	63 (9.78)
Highway	88 (13.66)
Min road	395 (61.34)
Side road	41 (6.37)
Rural road	47 (7.30)
Ring road	2 (0.31)
Dedicated road	4 (0.62)
Other	137 (17.6)
Organ affected	
Femur to the bottom	8 (1.0)
Shoulder joint down	3 (0.4)
Abdomen, chest, back and sternum	75 (9.6)
Neck and head	423 (54.4)
Road user type	
Pedestrian	107 (13.77)
Driver/rider	60 (7.72)
Passenger	606 (77.99)
Type of accident	
Traffic	728 (93.69)
Nontraffic	49 (6.31)
Final cause of death	
Head trauma	405 (52.12)
Bleeding	60 (7.72)
Multiple fractures	143 (18.40)
Burns	9 (1.16)
Suffocation	145 (18.66)
Other cases	15 (1.93)
Place of death	
Died at home	4 (0.5)
Died at hospital	258 (33.2)
Died at the scene	458 (58.9)
Died while being transferred to hospital	54 (6.9)
Unknown	3 (0.4)
Time of the accident	
Spring	183 (23.5)
Summer	248 (31.8)
Autumn	183 (23.5)
Winter	165 (21.2)
The vehicle used	
Agricultural vehicles	3 (0.4)
Bus	15 (1.9)
Truck	11 (1.4)
Personal car	544 (69.8)
Minibus	5 (0.6)
Motorcycle	31 (4.0)
Other vehicles	2 (0.3)
Unknown	8 (1.0)
Pickup truck	53 (6.8)
The vehicle involved (opposite)	
Agricultural vehicles	5 (0.6)
Bus	29 (3.7)
Truck	143 (18.4)
Personal car	220 (28.2)
Minibus	8 (1.0)
Motorcycles	4 (0.5)
Other vehicles	23 (3.0)
Unknown	19 (2.4)
Pickup truck	69 (8.9)
Nonvehicle involved	256 (32.9)

Abbreviation: RTA, road traffic accident.

Among the 777 fatal accidents involving women, 88 cases (11.32%) occurred on inner city roads, and 644 cases (82.88%) occurred on suburban roads. Out of these, 256 cases (32.9%) involved vehicle collisions with pedestrians.

### 3.2 Age segments and associated accident factors in RTA‐related fatalities among women of reproductive age

Table 2 displays the distribution of RTAs‐related mortality across various age groups among women of reproductive age. *χ* ^2^ test results revealed a significant difference in age, education (*p* < 0.0001), and road user types. Additionally, the Fischer test indicated significant differences in marital status (*p* < 0.0001), occupation (*p* < 0.0001), and type of accident (*p* < 0.0001) across different age groups. Across all age groups, injuries to the neck and head were the most common, followed by injuries to the abdomen, chest, back, and sternum. Traffic accidents were the predominant cause in most cases, and pedestrians constituted the largest group of RTA‐related victims in East Azerbaijan province. Among the 86 cases with femur‐to‐bottom trauma, only 2 cases exhibited pelvic injuries alone, while 47 cases (54.65%) had injuries to both the neck and head along with the pelvis. In 6 cases (6.98%), injuries were limited to the neck, while in 60 cases (69.77%), injuries encompassed the abdomen and chest. Additionally, 16 cases (18.6%) involved injuries to the hands and arms, 6 cases (6.98%) to the back and spinal cord, and 39 cases (45.35%) to the legs.

**Table 2:** The distribution of RTAs‐related mortality in different age groups of women of reproductive age.

	Different age groups of reproductive age women							
	15–19 years	20–24 years	25–29 years	30–34 years	35–39 years	40–44 years	45–49 years	*p* Value
Organ affected								<0.0001^a^
Femur to the bottom	7 (5.5)	14 (10.9)	26 (20.3)	24 (18.8)	23 (18)	17 (13.3)	17 (13.3)	
Shoulder joint down	2 (2.7)	9 (12.0)	12 (16.0)	14 (18.7)	16 (21.3)	11 (14.7)	11 (14.7)	
Abdomen, chest, back and sternum	18 (5.9)	38 (12.5)	54 (17.8)	54 (17.8)	46 (15.1)	45 (14.8)	49 (16.1)	
Neck and head	42 (6.4)	87 (13.3)	113 (17.3)	101 (15.5)	106 (16.3)	99 (15.2)	104 (16.0)	
Mechanism of the accident								<0.0001^a^
Rollover	19 (9.2)	32 (15.5)	37 (18.0)	28 (13.6)	34 (16.5)	28 (13.6)	28 (13.6)	
Vehicle collision with fixed agent	0 (0.0)	7 (10.6)	16 (24.2)	12 (18.2)	11 (16.7)	12 (18.2)	8 (12.1)	
Vehicle collision with pedestrians	7 (6.5)	9 (8.4)	15 (14.0)	13 (12.1)	18 (16.8)	20 (18.7)	25 (23.4)	
Vehicle collision with another vehicle	21 (5.5)	47 (12.4)	68 (17.9)	67 (17.6)	60 (15.8)	57 (15.8)	60 (15.8)	
Rode user type								0.02^a^
Pedestrian	7 (6.54)	9 (8.41)	15 (14.02)	13 (12.15)	18 (16.82)	20 (18.69)	25 (23.36)	
Driver/rider	0 (0.0)	4 (6.67)	8 (13.33)	18 (30.00)	12 (20.00)	10 (16.67)	8 (13.33)	
Passenger	40 (6.60)	86 (14.19)	114 (18.81)	93 (15.35	93 (15.35)	88 (14.52)	92 (15.18)	
Type of accident								0.99^b^
Traffic	45 (6.18)	92 (12.64)	129 (17.72)	117 (16.07)	117 (16.07)	109 (14.97)	119 (16.35)	
Nontraffic	3 (6.12)	7 (14.29)	8 (16.33)	7 (14.29)	8 (16.33)	9 (18.37)	7 (14.29)	
The main cause of death								<0.0001^a^
Bleeding	3 (5.00)	7 (11.67)	11 (18.33)	15 (25.00)	6 (10.00)	4 (6.67)	14 (23.33)	
Head trauma	29 (7.16)	53 (13.09)	73 (18.02)	62 (15.31)	63 (15.56)	55 (13.58)	70 (17.28)	
Mixed causes	9 (6.21)	19 (13.10)	22 (15.17)	26 (17.93)	24 (16.55)	28 (19.31)	17 (11.72)	
Multiple fractures	6 (4.20)	15 (10.49)	29 (20.28)	15 (10.49)	28 (19.58)	27 (18.88)	23 (16.08)	
Others	1 (4.17)	5 (20.83)	2 (8.33)	6 (25.00)	4 (16.67)	4 (16.67)	2 (8.33)	
Road type								0.47^a^
Freeway	0 (0.00)	9 (20.00)	9 (20.00)	6 (13.33)	7 (15.56)	5 (11.11)	9 (20.00)	
Highway	3 (5.00)	5 (8.33)	14 (23.33)	8 (13.33)	11 (18.33)	10 (16.67)	9 (15.00)	
Main road	14 (4.55)	41 (13.31)	47 (15.26)	55 (17.86)	56 (18.18)	45 (14.61)	50 (16.23)	
Side road	5 (15.63)	5 (15.63)	3 (9.38)	4 (12.50)	7 (21.88)	6 (18.75)	2 (6.25)	
Others	4 (9.52)	7 (16.67)	6 (14.29)	7 (16.67)	4 (9.52)	6 (14.29)	8 (19.05)	
Place of death								0.57^b^
Died at hospital	20 (7.75)	32 (12.40)	42 (16.28)	35 (13.57)	33 (12.79)	50 (19.38)	46 (17.83)	
Died at the scene	23 (5.01)	59 (12.85)	79 (17.21)	81 (17.65)	87 (18.95)	59 (12.85)	71 (15.47)	
Died while being transferred to hospital	5 (9.26)	8 (14.81)	14 (25.93)	9 (16.67)	5 (9.26)	8 (14.81)	5 (9.26)	
Unknown	0 (0.00)	0 (0.00)	2 (28.57)	0 (0.00)	0 (0.00)	1 (14.29)	4 (57.14)	

a Fisher's exact test.

b Pearson *χ* ^2^.

**Figure 1:**
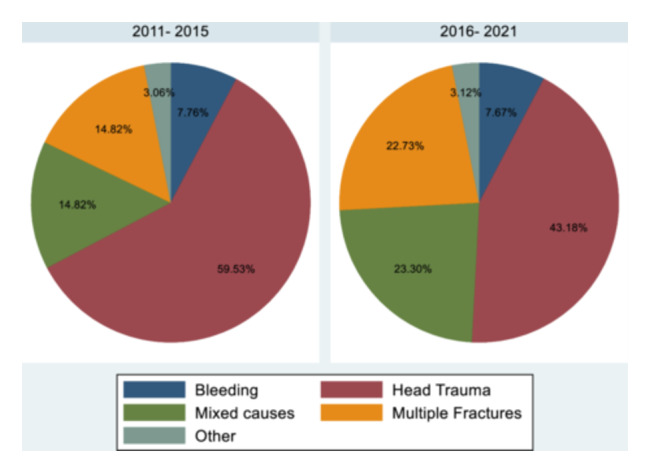
Type of accidents in 2011–2015 and 2016–2021 in percent. According to the *χ* ^2^ test, there was a significant difference in terms of the type of accidents between two time periods (*p* < 0.001). The mechanism of RTAs leading to the death of women of reproductive age in the last 5 years in comparison to the first 5 years of the decade. RTA, road traffic accident.

Employing the Fischer test, we observed a significant association between the location of the accident and the type of accident with the ultimate cause of death (*p* < 0.0001) (Figure 1). Various factors emerged as the primary causes of death on freeways, whereas head trauma prevailed as the leading cause of death on other types of roads. The Fischer test also revealed a significant relationship between the primary cause of death and the road type (*p* < 0.001) (see Table 3).

**Table 3:** The frequency (percent) of the main cause of death based on the effective variables on RTAs among women of reproductive age in the last two decades in East‐Azerbaijan province.

	Bleeding	Head Trauma	Mixed causes	Multiple fractures	Others	*p* Value^a^
Place of death						<0.0001
Died at hospital	29 (11.2)	122 (47.3)	42 (16.3)	56 (21.7)	9 (3.5)	
Died at the scene	24 (5.2)	259 (56.3)	87 (18.9)	76 (16.5)	14 (3.0)	
Died while being transferred to hospital	7 (13.0)	25 (46.3)	15 (27.8)	7 (13.0)	0 (0.0)	
Unknown	0 (0.0)	0 (0.0)	1 (14.3)	4 (57.1)	2 (28.6)	
Mechanism of the accident						<0.0001
Rollover	0 (0.0)	11 (55.0)	4 (20.0)	2 (10.0)	3 (15.0)	
Vehicle collision with fixed agent	20 (9.7)	106 (51.5)	38 (18.4)	40 (19.4)	2 (1.0)	
Vehicle collision with pedestrians	3 (4.5)	43 (65.2)	6 (9.1)	10 (15.2)	4 (6.1)	
Vehicle collision with another vehicle	7 (6.5)	58 (54.2)	24 (22.4)	14 (13.1)	4 (3.7)	
Others	30 (7.9)	188 (49.5)	73 (19.2)	77 (20.3)	12 (3.2)	
Vehicle involved (opposite)						<0.0001
Agricultural vehicles	0 (0.0)	3 (60.0)	1 (20.0)	1 (20.0)	0 (0.0)	
Bus	2 (6.9)	15 (51.7)	4 (13.8)	8 (27.6)	0 (0.0)	
Truck	22 (9.9)	115 (51.6)	40 (17.9)	37 (16.6)	9 (4.0)	
Personal car	22 (9.9)	115 (51.6)	40 (17.9)	37 (16.6)	9 (4.0)	
Minibus	0 (0.0)	5 (62.5)	3 (37.5)	0 (0.0)	0 (0.0)	
Motorcycles	1 (25.0)	3 (75.0)	0 (0.0)	0 (0.0)	0 (0.0)	
Other vehicles	0 (0.0)	17 (73.9)	1 (4.3)	5 (21.7)	0 (0.0)	
Unknown	0 (0.0)	11 (57.9)	4 (21.1)	3 (15.8)	1 (5.3)	
Pickup truck	1 (1.4)	34 (49.3)	15 (21.7)	16 (23.2)	3 (4.3)	
Nonvehicle involved	0 (0.0)	3 (60.0)	1 (20.0)	1 (20.0)	0 (0.0)	
Road type						<0.0001
Freeway	5 (11.1)	13 (28.9)	14 (31.1)	13 (28.9)	0 (0.0)	
Highway	2 (3.3)	35 (58.3)	12 (20.0)	11 (18.3)	0 (0.0)	
Main road	21 (6.8)	144 (46.8)	64 (20.8)	66 (21.4)	13 (4.2)	
Side road	5 (15.6)	13 (40.6)	6 (18.8)	7 (21.9)	1 (3.1)	
Others	4 (9.5)	24 (57.1)	3 (7.1)	8 (19.0)	3 (7.1)	

Abbreviation: RTA, road traffic accident.

a Fisher's exact test.

**Figure 2:**
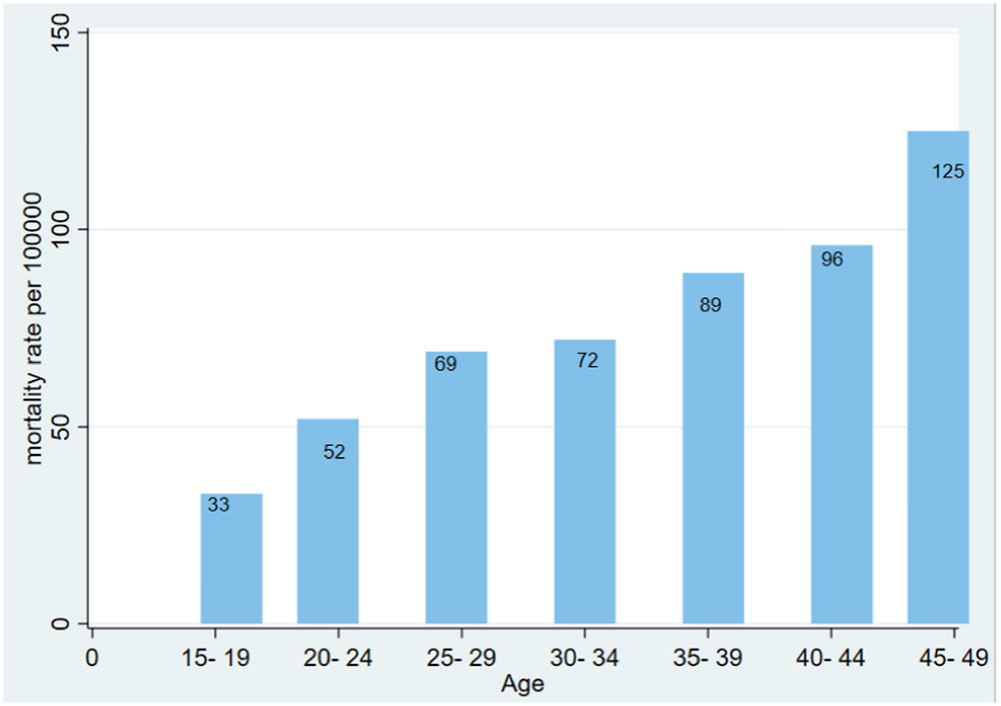
The mortality rate in different age groups of women of reproductive age per 100,000 individuals.

Table 4 and Figure 2 provide the RTAs‐related mortality rates for women of reproductive age, grouped in 5‐year increments from 15 to 49 years. In the 45‐49 age group, the mortality rate was reported as 125 cases per 100,000, and as age increases, so does the mortality rate.

**Table 4:** The rate of RTAs‐related mortality in women in the last two decades compared to the year 2016 in East Azerbaijan province.

Age group	2011–2021	The year 2016	Mortality rate per 100,000 for years
15–19	48	143,748	33
20–24	99	189,949	52
25–29	137	196,961	69
30–34	124	171,261	72
35–39	125	140,010	89
40–44	118	122,154	96
45–49	126	100,243	125

Abbreviation: RTA, road traffic accident.

## 4 DISCUSSION

There is a pressing need for epidemiological investigations aimed at delineating mortality patterns, identifying influential factors in traffic accidents, enhancing road safety cost‐effectively, and reducing road traffic injuries. In this current study, a significant proportion of reproductive‐age victims were vehicle riders. Following closely were pedestrians, exhibiting the highest mortality rate, a finding consistent with a study conducted in Georgia.^7^ This elevated mortality rate might be attributed to women's lesser use of motorcycles or bicycles and their reduced involvement in driving.^8^ The present investigation also revealed that working women had a lower incidence of RTAs‐related mortality compared to nonworking women, aligning with the observations in the Georgian study. However, the risk of road injuries in employed women is almost half that of unemployed women.^9^ Additionally, according to Lotfi et al.'s study, the highest number of road accidents was among housekeepers.^8^

The literature suggests that the rate of road accidents is notably higher in the 25–29 age group, possibly due to risky behaviors, lack of experience, and peer influence.^10^, ^11^, ^12^ In our present study, a higher incidence of road accidents was observed among married individuals, though previous research did not establish a significant correlation between marital status and RTAs. Some studies have reported that married women face a mortality risk twice as high as never‐married women due to higher alcohol consumption and lower seat belt usage.^13^, ^14^ Limited epidemiological studies are available on this subject.^15^, ^16^

Individuals with lower education levels exhibited the highest rate of RTAs‐related mortality in our study. Braver et al. found that the relative risk of mortality was 2.8 times higher in women with lower education compared to educated individuals.^17^ Other study reported a 1.6 times higher relative risk of mortality in women aged 20–64 for the years 1984–1997.^18^ Cubbin, in a study on people aged 18–64 who died between 1987 and 1995, utilizing the Survey and National Mortality Index, also discovered a higher relative risk of mortality among women with lower education compared to those with a diploma or higher.^16^ Beyond vehicle safety concerns, less‐educated individuals may face higher RTAs risk due to limited knowledge of driving rules and traffic signs.^16^, ^17^, ^18^

Our study revealed a significant difference in the type of road user at the time of the accident, with passengers experiencing the highest mortality rate across all age groups. Globally, vulnerable road users, including pedestrians, make up half of all RTA‐related mortality cases.^5^ Studies indicate that pedestrians in the 17–30 age group, regardless of gender, often violate traffic rules, increasing the risk of mortality in this age bracket. Similar research reported higher mortality rates among pedestrian women compared to other women and noted a 50% higher risk in women compared to men.^19^

The rate of RTAs‐related mortality significantly varied based on the type of road, with higher mortality rates observed on main roads across all age groups. This finding aligns with the categorization of urban areas as high‐risk and vulnerable in terms of traffic by FSU.^16^, ^20^, ^21^ However, some studies identify rural roads as high‐risk due to factors like poor traffic control, inadequate road safety infrastructure (such as insufficient lighting, lack of sidewalks, and guardrails), and inadequate postaccident aid. Another study highlighted issues among agricultural vehicle users, including insufficient driving training, absence of personal protective equipment, low literacy levels, and young age. Implementing safety promotion programs, upgrading agricultural equipment, and promoting safety through protective measures can help reduce the mortality rate in road accidents.^22^

In all age groups, head trauma emerged as the primary cause of death among women of reproductive age from 2011 to 2021. This finding is consistent with Sadeghi et al.'s research, which reported head trauma as the primary cause of death in 51.3% of cases among farmers involved in fatal road accidents.^22^

The most prevalent mechanism of accidents in our study was vehicle collision with other vehicles, in contrast to Sadeghi et al.'s findings, which highlighted rollovers as the primary cause.^23^ The International Community for Agricultural Safety and Health has also identified tractor rollovers as the main cause of death among farmers.^24^

## 5 CONCLUSION

In conclusion, our study sheds light on various factors influencing RTAs and their associated mortalities among women of reproductive age. Furthermore, the stark correlation between lower education levels and RTAs‐related mortality underscores the importance of education in promoting road safety. The risk factors associated with less‐educated individuals extend beyond vehicle safety concerns, encompassing limited knowledge of traffic rules and signs. Our observation of passengers experiencing the highest mortality rate emphasizes the vulnerability of road users, particularly pedestrians, in traffic accidents. Pedestrian violations in the 17–30 age group further emphasize the importance of education and awareness campaigns aimed at reducing risky behavior. The discrepancy in mortality rates between main roads and rural roads highlights the need for tailored safety measures in different settings. Safety promotion programs, infrastructure improvements, and protective measures, especially among vulnerable agricultural vehicle users, hold the potential to mitigate RTAs and fatalities. Finally, the predominance of head trauma as the primary cause of death underscores the critical role of head injury prevention strategies. In sum, our findings contribute to the body of knowledge needed to develop effective interventions and policies to enhance road safety and reduce RTAs‐related mortality among women of reproductive age.

## AUTHOR CONTRIBUTIONS

**Mina Golestani**: investigation; writing—original draft; methodology; validation; data curation; resources. **Homayoun Sadeghi‐Bazargani**: Validation; methodology; conceptualization; investigation; funding acquisition; resources. **Leili Faraji Gavgani**: Conceptualization; investigation; writing—original draft; methodology; validation; formal analysis; data curation. **Roghayeh Khabiri**: Investigation; methodology; data curation; validation. **Leila Jahangiry**: Conceptualization; investigation; writing—review and editing; supervision; formal analysis.

## CONFLICT OF INTEREST STATEMENT

The authors declare no conflict of interest.

## ETHICS STATEMENT

The current study was approved by the Committee of Ethics of Tabriz University of Medical Sciences (IR.TBZMED.REC.1401.289) and conducted according to the ethical norms and guidelines. The authors also confirmed the ethical instructions were implemented in method.

## TRANSPARENCY STATEMENT

The lead author Leila Jahangiry affirms that this manuscript is an honest, accurate, and transparent account of the study being reported; that no important aspects of the study have been omitted; and that any discrepancies from the study as planned (and, if relevant, registered) have been explained.

## Data Availability

The data collection tools and datasets generated and/or analyzed during the current study are available from the corresponding author upon reasonable request.
